# The role of gender on academic performance in STEM-related disciplines: Data from a tertiary institution

**DOI:** 10.1016/j.dib.2018.03.052

**Published:** 2018-03-17

**Authors:** Temitope M. John, Joke A. Badejo, Segun I. Popoola, David O. Omole, Jonathan A. Odukoya, Priscilla O. Ajayi, Mary Aboyade, Aderemi A. Atayero

**Affiliations:** a Department of Electrical and Information Engineering, Covenant University, Ota, Ogun State, Nigeria; b Department of Civil Engineering, Covenant University, Ota, Ogun State, Nigeria; c Department of Psychology, Covenant University, Ota, Ogun State, Nigeria; d Center for Systems and Information Services, Covenant University, Ota, Ogun State, Nigeria; e Covenant University Data Analytics Cluster, Covenant University, Ota, Ogun State, Nigeria

**Keywords:** Learning analytics, STEM students, STEM, STEM education, Gender roles, Undergraduates, Education data mining, Smart campus, Nigerian university

## Abstract

This data article presents data of academic performances of undergraduate students in Science, Technology, Engineering and Mathematics (STEM) disciplines in Covenant University, Nigeria. The data shows academic performances of Male and Female students who graduated from 2010 to 2014. The total population of samples in the observation is 3046 undergraduates mined from Biochemistry (BCH), Building technology (BLD), Computer Engineering (CEN), Chemical Engineering (CHE), Industrial Chemistry (CHM), Computer Science (CIS), Civil Engineering (CVE), Electrical and Electronics Engineering (EEE), Information and Communication Engineering (ICE), Mathematics (MAT), Microbiology (MCB), Mechanical Engineering (MCE), Management and Information System (MIS), Petroleum Engineering (PET), Industrial Physics-Electronics and IT Applications (PHYE), Industrial Physics-Applied Geophysics (PHYG) and Industrial Physics-Renewable Energy (PHYR). The detailed dataset is made available in form of a Microsoft Excel spreadsheet in the supplementary material of this article.

**Specifications Table**

**Table:** 

Subject area	*Engineering Education*
More specific subject area	*Learning Analytics*
Type of data	*Table, figures, excel file, and graphs*
How data was acquired	*The paper presents a five-year study period of STEM programs, Gender, Secondary School Grade Point Average (SGPA), Overall Cumulative Grade Point Average (CGPA), Cumulative Grade Point Average at the end of first year (CGPA 100), Cumulative Grade Point Average at the end of second year (CGPA 200), Cumulative Grade Point Average at the end of third year (CGPA 300), Cumulative Grade Point Average at the end of fourth year (CGPA 400)*
Data format	*Raw, analyzed*
Experimental factors	*Only undergraduates with complete records were included in this study*
Experimental features	*The paper includes descriptive statistics and box-plots for the 17 programs under the observation*
Data source location	*The data was gathered from the department of students records at Covenant University, Ota, Nigeria (Latitude 6.67181*°*N, Longitude 3.1581*° *E)*
Data accessibility	*Data is within this article in the supplementary materials section*

**Value of the data**

• The empirical data provided will insights to academic performances of male and female students in STEM programs.
• Provides corroborative data to the underrepresentation of males in the social sciences and the underrepresentation of female students in the physical sciences [1], [2].
• Data provided could provide answers to STEM disciplines favored by male and female students [3].
• To encourage evidence research in student educational mining especially with regards to gender roles in developing countries and smart campuses [4], [5].

## 1 Data

Gender is perceived to affect students’ academic performance at different levels of academic pursuit. In [2], a study was conducted which concluded that Teachers and Students attributed Masculine characteristics to the Science professional and Feminine Characteristics to the humanities. They also believe that Male students’ outperformed female students in STEM related disciplines [6], [7], [8].

The population sample in this data consists of undergraduate students who graduated from STEM majors between 2010 and 2014 in Covenant University, Ota, Nigeria. The data was retrieved from the department of students’ record. A total of 3046 undergraduate were sampled from Biochemistry (BCH), Building technology (BLD), Computer Engineering (CEN), Chemical Engineering (CHE), Industrial Chemistry (CHM), Computer Science (CIS), Civil Engineering (CVE), Electrical and Electronics Engineering (EEE), Information and Communication Engineering (ICE), Mathematics (MAT), Microbiology (MCB), Mechanical Engineering (MCE), Management and Information System (MIS), Petroleum Engineering (PET),Industrial Physics-Electronics and IT Applications (PHYE), Industrial Physics-Applied Geophysics (PHYG) and Industrial Physics-Renewable Energy (PHYR). The descriptive statistics for male and female students in STEM programs showing mean, median, mode, standard deviation, variance, maximum, minimum, range and total number of samples is given in Table 1, Table 2, Table 3, Table 4, Table 5, Table 6. The data shows Secondary School Grade Point Average (SGPA), Cumulative Grade Point Average at the end of the first year (CGPA100), Cumulative Grade Point Average at the end of the second year (CGPA200), Cumulative Grade Point Average at the end of the third year (CGPA300), Cumulative Grade Point Average at the end of the fourth year (CGPA400) and Overall Cumulative Grade Point Average (Overall CGPA).

**Table 1:** **Descriptive statistics of SGPA of female and male students from 2010–2014**.

Program code	Gender	Mean	Median	Mode	Standard deviation	Variance	Maximum	Minimum	Range	Total N
BCH	Female	3.19	3.13	3.13	0.58	0.33	4.45	1.76	2.69	113
	Male	2.94	2.95	3.13	0.43	0.19	3.83	2.08	1.75	35
										
BLD	Female	2.89	2.94	3.04	0.63	0.40	4.31	1.74	2.57	30
	Male	2.76	2.71	2.50	0.51	0.26	4.45	1.47	2.98	67
										
CEN	Female	3.32	3.35	2.50	0.63	0.40	4.77	1.67	3.10	72
	Male	3.22	3.21	2.73	0.51	0.26	4.58	2.14	2.44	165
										
CHE	Female	3.31	3.34	2.86	0.64	0.41	4.51	1.77	2.74	78
	Male	3.35	3.28	3.13	0.60	0.36	4.88	2.10	2.78	135
										
CHM	Female	2.93	2.89	2.50	0.54	0.29	4.30	1.74	2.56	62
	Male	2.91	2.89	3.13	0.54	0.29	4.06	1.95	2.11	49
										
CIS	Female	3.08	3.05	3.75	0.61	0.37	4.53	1.76	2.77	120
	Male	3.03	3.01	2.81	0.59	0.35	4.93	1.77	3.16	222
										
CVE	Female	3.07	2.97	2.97	0.59	0.35	4.38	1.88	2.50	24
	Male	2.96	2.92	3.05	0.57	0.33	4.44	1.72	2.72	143
										
EEE	Female	3.50	3.67	3.75	0.64	0.41	4.88	2.19	2.69	81
	Male	3.36	3.30	3.13	0.62	0.38	4.77	1.96	2.81	337
										
ICE	Female	3.26	3.33	3.13	0.55	0.30	4.38	2.05	2.33	95
	Male	3.06	3.05	2.50	0.59	0.35	4.38	1.74	2.64	150
										
MAT	Female	2.74	2.58	2.50	0.60	0.35	4.13	1.88	2.25	27
	Male	2.83	2.62	2.50	0.67	0.45	4.38	2.03	2.35	34
										
MCB	Female	3.04	2.97	3.13	0.55	0.31	4.30	1.65	2.65	130
	Male	3.10	3.05	2.92	0.56	0.31	4.30	2.05	2.25	34
										
MCE	Female	3.67	3.96	4.38	0.81	0.66	4.53	2.00	2.53	16
	Male	3.28	3.30	3.13	0.57	0.32	4.64	1.88	2.76	168
										
MIS	Female	2.89	2.86	2.50	0.58	0.33	4.30	1.65	2.65	151
	Male	2.73	2.71	2.50	0.55	0.30	3.98	1.46	2.52	156
										
PET	Female	3.31	3.36	3.13	0.62	0.39	4.53	1.95	2.58	70
	Male	3.19	3.13	2.99	0.58	0.34	4.45	1.99	2.46	137
										
PHYE	Female	3.00	3.19	2.14	0.57	0.33	4.14	2.14	2.00	13
	Male	2.83	2.77	2.99	0.50	0.25	3.91	1.83	2.08	69
										
PHYG	Female	3.08	3.20	2.42	0.44	0.20	3.52	2.42	1.10	7
	Male	3.03	2.98	2.89	0.68	0.46	4.38	1.67	2.71	30
										
PHYR	Female	2.99	3.06	2.19	0.69	0.48	3.66	2.19	1.47	4
	Male	2.98	2.84	1.67	0.83	0.68	4.51	1.67	2.84	22

**Table 2:** Descriptive statistics of CGPA 100 for female and male students from 2010–2014.

Program code	Gender	Mean	Median	Mode	Standard deviation	Variance	Maximum	Minimum	Range	Total N
BCH	Female	3.58	3.62	3.85	0.63	0.40	5.00	1.73	3.27	113
	Male	3.26	3.30	3.30	0.54	0.29	4.43	1.78	2.65	35
										
BLD	Female	3.16	3.22	3.20	0.59	0.35	4.43	2.02	2.41	30
	Male	3.00	3.00	2.61	0.52	0.27	4.37	1.98	2.39	67
										
CEN	Female	4.00	4.02	3.57	0.49	0.24	4.93	3.02	1.91	72
	Male	3.71	3.78	3.98	0.61	0.38	4.84	1.92	2.92	165
										
CHE	Female	4.00	4.16	4.43	0.68	0.46	4.89	2.33	2.56	78
	Male	3.96	4.06	3.79	0.59	0.35	4.91	1.95	2.96	135
										
CHM	Female	3.15	3.13	1.82	0.72	0.52	4.60	1.59	3.01	62
	Male	3.44	3.50	2.63	0.64	0.41	4.73	2.17	2.56	49
										
CIS	Female	3.65	3.63	3.38	0.63	0.40	4.96	1.93	3.03	120
	Male	3.56	3.53	3.51	0.63	0.40	4.91	1.83	3.08	222
										
CVE	Female	3.75	3.76	3.51	0.55	0.31	4.80	2.25	2.55	24
	Male	3.60	3.58	4.02	0.63	0.39	4.96	1.60	3.36	143
										
EEE	Female	4.15	4.22	4.11	0.58	0.33	4.93	1.71	3.22	81
	Male	4.01	4.11	4.13	0.54	0.29	4.94	2.24	2.70	337
										
ICE	Female	3.73	3.80	3.87	0.58	0.34	4.96	2.32	2.64	95
	Male	3.73	3.73	3.51	0.57	0.33	4.80	2.30	2.50	150
										
MAT	Female	3.65	3.65	3.52	0.69	0.48	4.67	1.72	2.95	27
	Male	3.14	2.99	2.70	0.57	0.32	4.23	2.00	2.23	34
										
MCB	Female	3.36	3.45	2.59	0.64	0.41	4.70	1.65	3.05	130
	Male	3.32	3.26	3.15	0.57	0.33	4.37	2.26	2.11	34
										
MCE	Female	4.22	4.26	3.93	0.47	0.22	4.87	3.18	1.69	16
	Male	3.89	3.97	4.29	0.59	0.35	4.87	2.20	2.67	168
										
MIS	Female	3.19	3.24	2.68	0.65	0.42	4.52	1.57	2.95	151
	Male	2.97	3.00	2.23	0.58	0.33	4.30	1.64	2.66	156
										
PET	Female	3.92	3.89	3.78	0.58	0.34	4.93	2.55	2.38	70
	Male	3.80	3.85	4.22	0.64	0.41	4.89	1.64	3.25	137
										
PHYE	Female	3.42	3.63	3.63	0.73	0.53	4.31	1.98	2.33	13
	Male	3.45	3.53	3.53	0.57	0.32	4.40	1.80	2.60	69
										
PHYG	Female	3.84	3.78	3.27	0.43	0.18	4.39	3.27	1.12	7
	Male	3.36	3.34	3.76	0.56	0.32	4.29	2.35	1.94	30
										
PHYR	Female	3.47	3.67	2.53	0.65	0.43	4.00	2.53	1.47	4
	Male	3.53	3.48	3.27	0.53	0.28	4.36	2.80	1.56	22

**Table 3:** Descriptive statistics of CGPA 200 for female and male students from 2010–2014.

Program code	Gender	Mean	Median	Mode	Standard deviation	Variance	Maximum	Minimum	Range	Total N
BCH	Female	3.58	3.61	4.06	0.76	0.58	4.98	1.84	3.14	113
	Male	3.08	2.98	2.85	0.62	0.39	4.30	1.94	2.36	35
										
BLD	Female	3.37	3.43	2.89	0.67	0.44	4.62	2.18	2.44	30
	Male	2.93	3.00	1.88	0.77	0.59	4.47	1.23	3.24	67
										
CEN	Female	3.58	3.56	3.92	0.60	0.36	4.90	2.17	2.73	72
	Male	3.18	3.18	3.20	0.74	0.54	4.86	1.44	3.42	165
										
CHE	Female	3.58	3.71	3.71	0.75	0.56	4.74	1.54	3.20	78
	Male	3.44	3.43	3.37	0.72	0.52	4.88	1.87	3.01	135
										
CHM	Female	3.40	3.47	2.03	0.79	0.63	4.76	1.57	3.19	62
	Male	3.41	3.54	3.02	0.76	0.58	4.83	1.76	3.07	49
										
CIS	Female	3.73	3.77	3.14	0.74	0.55	5.00	1.98	3.02	120
	Male	3.19	3.23	2.22	0.84	0.70	4.98	1.42	3.56	222
										
CVE	Female	3.36	3.34	2.96	0.58	0.34	4.33	1.70	2.63	24
	Male	3.00	2.93	2.36	0.74	0.54	4.92	1.61	3.31	143
										
EEE	Female	3.79	3.78	4.00	0.67	0.45	4.92	1.88	3.04	81
	Male	3.43	3.44	4.10	0.76	0.57	4.90	1.34	3.56	337
										
ICE	Female	3.31	3.29	3.29	0.75	0.56	4.88	1.53	3.35	95
	Male	3.21	3.20	3.06	0.71	0.51	4.90	1.53	3.37	150
										
MAT	Female	3.88	3.87	4.13	0.65	0.43	4.89	2.42	2.47	27
	Male	2.76	2.78	2.76	0.80	0.64	4.23	1.21	3.02	34
										
MCB	Female	3.21	3.29	3.94	0.79	0.62	4.71	1.72	2.99	130
	Male	2.99	2.90	2.55	0.86	0.74	4.65	1.72	2.93	34
										
MCE	Female	3.90	3.96	3.92	0.62	0.38	4.71	2.36	2.35	16
	Male	3.31	3.29	2.70	0.73	0.53	4.87	1.55	3.32	168
										
MIS	Female	3.60	3.63	4.07	0.78	0.61	4.88	1.26	3.62	151
	Male	2.89	2.90	2.64	0.77	0.60	4.69	1.17	3.52	156
										
PET	Female	3.38	3.53	3.33	0.75	0.57	4.71	1.69	3.02	70
	Male	3.19	3.13	3.04	0.68	0.46	4.96	1.63	3.33	137
										
PHYE	Female	3.65	3.84	3.84	0.84	0.71	4.59	1.80	2.79	13
	Male	3.20	3.24	2.77	0.76	0.57	4.53	1.30	3.23	69
										
PHYG	Female	3.89	4.23	2.31	0.87	0.75	4.70	2.31	2.39	7
	Male	3.03	2.96	2.22	0.81	0.65	4.45	1.41	3.04	30
										
PHYR	Female	3.51	3.58	2.61	0.73	0.53	4.27	2.61	1.66	4
	Male	3.40	3.34	3.25	0.80	0.64	4.70	1.77	2.93	22

**Table 4:** Descriptive statistics of CGPA 300 for female and male students from 2010–2014.

Program code	Gender	Mean	Median	Mode	Standard deviation	Variance	Maximum	Minimum	Range	Total N
BCH	Female	3.61	3.68	3.94	0.74	0.55	5.00	1.97	3.03	113
	Male	3.16	3.28	2.13	0.76	0.58	4.94	1.49	3.45	35
										
BLD	Female	3.76	3.79	3.91	0.61	0.37	4.91	2.54	2.37	30
	Male	3.20	3.25	2.19	0.82	0.68	4.82	1.41	3.41	67
										
CEN	Female	3.82	3.94	3.66	0.73	0.53	4.87	1.42	3.45	72
	Male	3.22	3.40	3.83	0.98	0.97	4.84	0.63	4.21	165
										
CHE	Female	3.65	3.87	3.87	0.82	0.67	4.85	1.47	3.38	78
	Male	3.35	3.43	3.13	0.82	0.68	4.94	1.27	3.67	135
										
CHM	Female	3.82	3.97	4.13	0.68	0.47	4.77	2.10	2.67	62
	Male	3.86	3.87	3.55	0.48	0.23	4.87	2.81	2.06	49
										
CIS	Female	3.71	3.82	3.90	0.81	0.65	5.00	1.34	3.66	120
	Male	3.29	3.35	3.48	0.89	0.79	4.93	0.97	3.96	222
										
CVE	Female	3.75	3.92	3.86	0.63	0.39	4.67	2.51	2.16	24
	Male	3.13	3.19	2.76	0.93	0.87	4.93	0.99	3.94	143
										
EEE	Female	3.91	4.09	4.19	0.77	0.59	4.98	1.10	3.88	81
	Male	3.47	3.60	3.96	0.87	0.76	4.89	1.05	3.84	337
										
ICE	Female	3.57	3.62	4.60	0.89	0.80	4.81	1.09	3.72	95
	Male	3.15	3.26	3.02	0.92	0.85	4.98	1.23	3.75	150
										
MAT	Female	3.93	4.03	3.97	0.64	0.41	4.86	2.45	2.41	27
	Male	3.01	2.97	2.29	0.76	0.57	4.48	1.73	2.75	34
										
MCB	Female	3.81	4.02	4.62	0.80	0.64	4.86	1.64	3.22	130
	Male	3.61	3.57	2.52	0.83	0.68	4.93	1.77	3.16	34
										
MCE	Female	3.81	3.94	4.11	0.66	0.44	4.52	1.74	2.78	16
	Male	3.06	3.00	3.13	0.86	0.74	4.98	1.31	3.67	168
										
MIS	Female	3.56	3.74	3.78	0.80	0.64	4.93	0.87	4.06	151
	Male	3.04	3.07	3.07	0.80	0.65	4.74	0.83	3.91	156
										
PET	Female	3.47	3.51	2.98	0.71	0.51	4.72	2.10	2.62	70
	Male	3.17	3.26	3.17	0.77	0.59	4.83	1.18	3.65	137
										
PHYE	Female	3.98	4.24	2.94	0.75	0.57	4.86	2.58	2.28	13
	Male	3.38	3.38	3.83	0.57	0.32	4.66	1.76	2.90	69
										
PHYG	Female	4.08	4.29	2.97	0.61	0.37	4.65	2.97	1.68	7
	Male	3.13	3.10	2.94	0.54	0.29	4.16	2.08	2.08	30
										
PHYR	Female	3.71	3.68	3.42	0.27	0.07	4.06	3.42	0.64	4
	Male	3.43	3.37	4.26	0.56	0.31	4.45	2.45	2.00	22

**Table 5:** Descriptive statistics of CGPA 400 for female and male students from 2010–2014.

Program code	Gender	Mean	Median	Mode	Standard deviation	Variance	Maximum	Minimum	Range	Total N
BCH	Female	3.80	3.93	4.51	0.77	0.60	5.00	1.74	3.26	113
	Male	3.34	3.28	2.35	0.66	0.44	4.73	2.22	2.51	35
										
BLD	Female	3.77	3.91	4.31	0.66	0.44	4.77	2.06	2.71	30
	Male	3.18	3.15	2.67	0.76	0.57	4.65	1.39	3.26	67
										
CEN	Female	3.98	4.09	4.38	0.69	0.48	4.90	1.82	3.08	72
	Male	3.46	3.66	3.79	0.82	0.67	4.90	.60	4.30	165
										
CHE	Female	3.86	4.06	3.53	0.78	0.61	4.91	1.97	2.94	78
	Male	3.65	3.78	4.06	0.90	0.80	4.97	1.00	3.97	135
										
CHM	Female	3.84	3.95	4.09	0.74	0.54	4.86	1.57	3.29	62
	Male	3.82	3.87	3.57	0.55	0.30	4.93	2.57	2.36	49
										
CIS	Female	3.75	3.79	4.40	0.73	0.54	5.00	1.42	3.58	120
	Male	3.26	3.33	3.93	0.79	0.62	4.88	1.54	3.34	222
										
CVE	Female	4.19	4.22	4.17	0.55	0.30	4.93	2.97	1.96	24
	Male	3.60	3.70	4.17	0.81	0.65	4.97	1.55	3.42	143
										
EEE	Female	3.75	3.79	3.10	0.69	0.48	4.77	1.40	3.37	81
	Male	3.38	3.44	3.48	0.77	0.59	5.00	1.26	3.74	337
										
ICE	Female	3.74	3.76	3.76	0.69	0.47	4.93	1.67	3.26	95
	Male	3.33	3.48	3.52	0.80	0.64	4.90	1.06	3.84	150
										
MAT	Female	3.96	3.93	3.50	0.62	0.39	4.86	2.67	2.19	27
	Male	2.95	3.04	2.29	0.72	0.52	4.26	1.84	2.42	34
										
MCB	Female	3.84	4.03	4.23	0.76	0.58	4.95	1.87	3.08	130
	Male	3.65	3.70	1.58	0.85	0.73	4.96	1.58	3.38	34
										
MCE	Female	3.97	4.02	4.00	0.45	0.20	4.55	3.07	1.48	16
	Male	3.52	3.65	4.55	0.79	0.63	4.97	1.25	3.72	168
										
MIS	Female	3.63	3.71	3.89	0.74	0.55	4.87	1.58	3.29	151
	Male	3.01	2.99	2.93	0.78	0.61	4.52	1.34	3.18	156
										
PET	Female	3.67	3.66	3.54	0.69	0.47	5.00	2.23	2.77	70
	Male	3.40	3.41	3.61	0.75	0.56	4.89	0.00	4.89	137
										
PHYE	Female	4.05	4.48	2.37	0.95	0.89	4.91	2.37	2.54	13
	Male	3.40	3.44	4.00	0.74	0.55	4.67	1.75	2.92	69
										
PHYG	Female	4.09	4.28	4.63	0.66	0.44	4.63	2.73	1.90	7
	Male	3.04	3.05	3.42	0.78	0.61	4.44	1.63	2.81	30
										
PHYR	Female	3.74	3.61	2.90	0.84	0.70	4.86	2.90	1.96	4
	Male	3.48	3.52	4.03	0.70	0.50	4.60	1.82	2.78	22

**Table 6:** Descriptive statistics of overall CGPA for female and male students from 2010–2014.

Program	Gender	Mean	Median	Mode	Standard deviation	Variance	Maximum	Minimum	Range	Total N
BCH	Female	3.64	3.72	3.26	0.68	0.46	4.99	1.91	3.08	113
	Male	3.22	3.17	2.63	0.53	0.28	4.33	2.21	2.12	35
										
BLD	Female	3.65	3.64	4.06	0.54	0.29	4.67	2.51	2.16	30
	Male	3.19	3.11	1.81	0.65	0.42	4.56	1.81	2.75	67
										
CEN	Female	3.86	3.83	3.49	0.54	0.30	4.78	2.16	2.62	72
	Male	3.37	3.48	3.21	0.71	0.51	4.67	1.84	2.83	165
										
CHE	Female	3.80	3.99	3.61	0.66	0.44	4.86	2.04	2.82	78
	Male	3.59	3.67	3.43	0.67	0.45	4.83	1.94	2.89	135
										
CHM	Female	3.52	3.56	3.36	0.67	0.45	4.66	1.79	2.87	62
	Male	3.61	3.66	3.01	0.57	0.32	4.83	2.52	2.31	49
										
CIS	Female	3.71	3.77	3.27	0.67	0.45	4.99	1.87	3.12	120
	Male	3.34	3.37	2.55	0.71	0.51	4.84	1.90	2.94	222
										
CVE	Female	3.85	3.90	3.97	0.48	0.23	4.68	2.81	1.87	24
	Male	3.40	3.42	3.76	0.70	0.49	4.93	1.97	2.96	143
										
EEE	Female	3.94	4.01	3.41	0.61	0.38	4.87	1.73	3.14	81
	Male	3.57	3.63	3.28	0.68	0.46	4.85	1.74	3.11	337
										
ICE	Female	3.66	3.66	3.77	0.67	0.45	4.89	1.80	3.09	95
	Male	3.40	3.46	2.96	0.68	0.47	4.76	1.85	2.91	150
										
MAT	Female	3.90	4.03	3.43	0.62	0.38	4.80	2.41	2.39	27
	Male	2.97	2.96	2.90	0.63	0.39	4.27	1.91	2.36	34
										
MCB	Female	3.52	3.69	3.98	0.69	0.48	4.70	1.79	2.91	130
	Male	3.35	3.28	2.92	0.72	0.52	4.63	2.07	2.56	34
										
MCE	Female	4.03	4.05	2.65	0.47	0.22	4.65	2.65	2.00	16
	Male	3.49	3.53	3.95	0.66	0.44	4.88	1.99	2.89	168
										
MIS	Female	3.50	3.56	4.11	0.67	0.45	4.71	1.72	2.99	151
	Male	2.99	3.02	2.45	0.64	0.41	4.51	1.52	2.99	156
										
PET	Female	3.67	3.67	3.22	0.62	0.39	4.80	2.42	2.38	70
	Male	3.44	3.43	3.83	0.59	0.35	4.85	2.07	2.78	137
										
PHYE	Female	3.76	4.01	4.01	0.79	0.62	4.50	2.31	2.19	13
	Male	3.35	3.41	3.55	0.61	0.37	4.38	1.80	2.58	69
										
PHYG	Female	3.95	4.18	2.78	0.63	0.39	4.56	2.78	1.78	7
	Male	3.14	3.18	3.62	0.64	0.41	4.27	1.88	2.39	30
										
PHYR	Female	3.58	3.48	3.09	0.58	0.33	4.28	3.09	1.19	4
	Male	3.45	3.43	3.53	0.63	0.39	4.46	2.13	2.33	22

## 2 Experimental design, materials, and methods

**Fig. 1:**
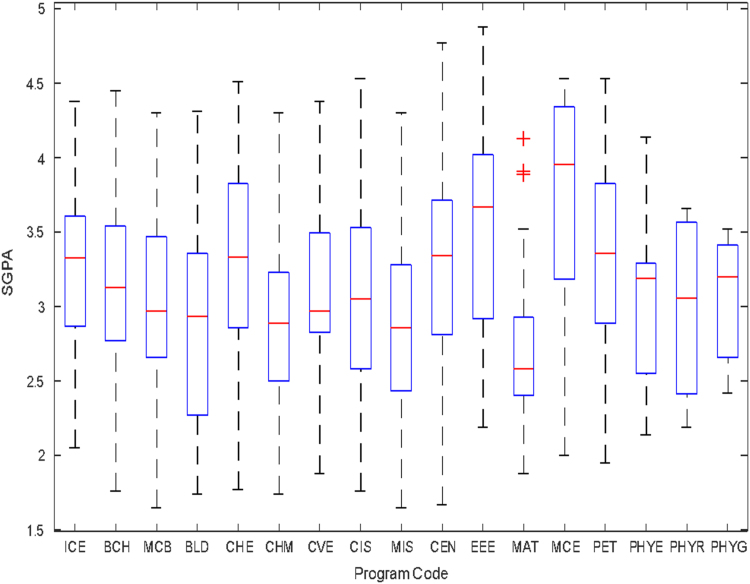
Boxplot of SGPA data for Female STEM students (2010–2014).

**Fig. 2:**
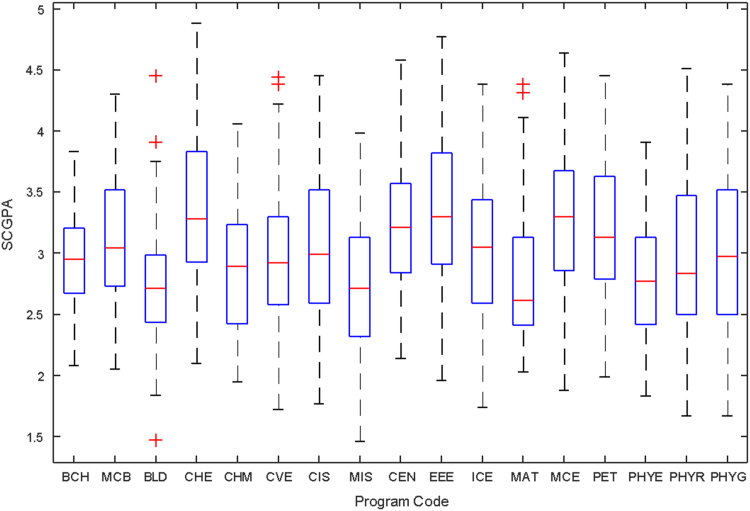
Boxplot of SGPA data for Male STEM students (2010–2014).

**Fig. 3:**
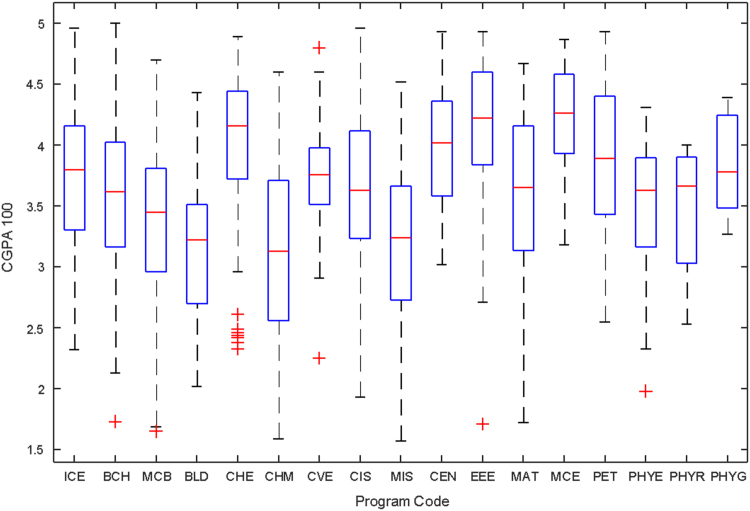
Boxplot of CGPA100 data for Female STEM students (2010–2014).

**Fig. 4:**
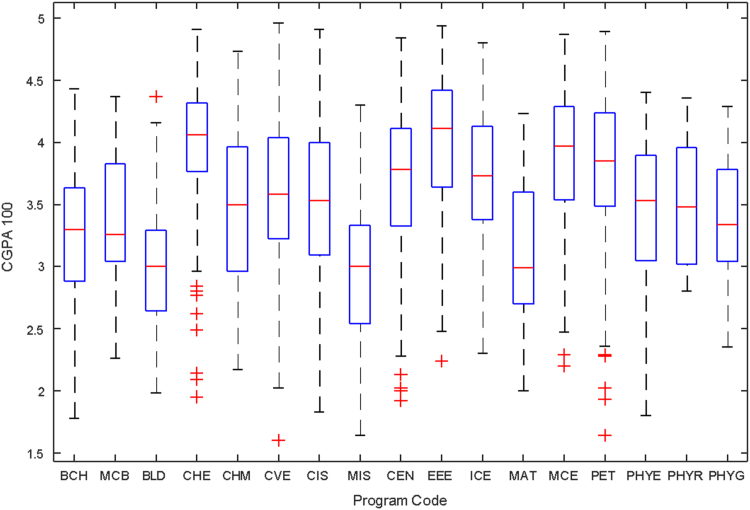
Boxplot of CGPA100 data for Male STEM students (2010–2014).

**Fig. 5:**
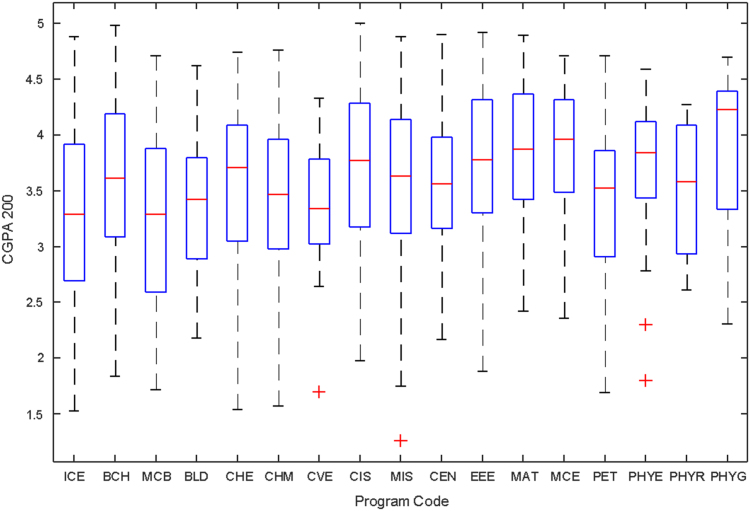
Boxplot of CGPA200 data for Female STEM students (2010–2014).

**Fig. 6:**
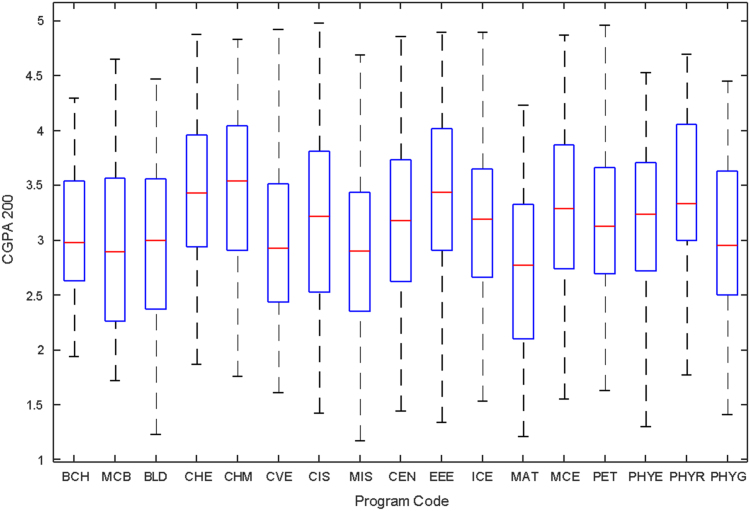
Boxplot of CGPA200 data for Male STEM students (2010–2014).

**Fig. 7:**
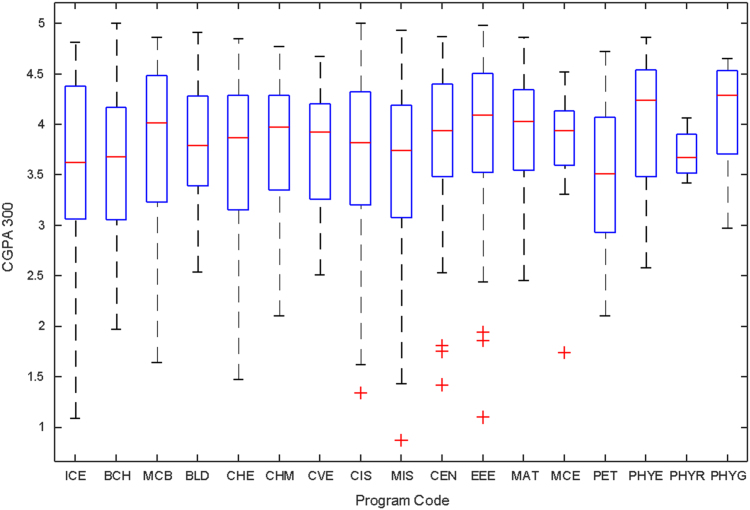
Boxplot of CGPA300 data for Female STEM students (2010–2014).

**Fig. 8:**
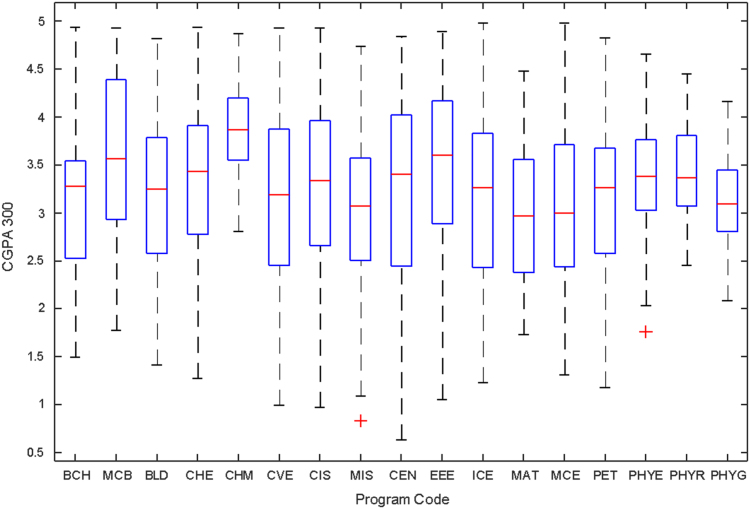
Boxplot of CGPA300 data for Male STEM students (2010–2014).

**Fig. 9:**
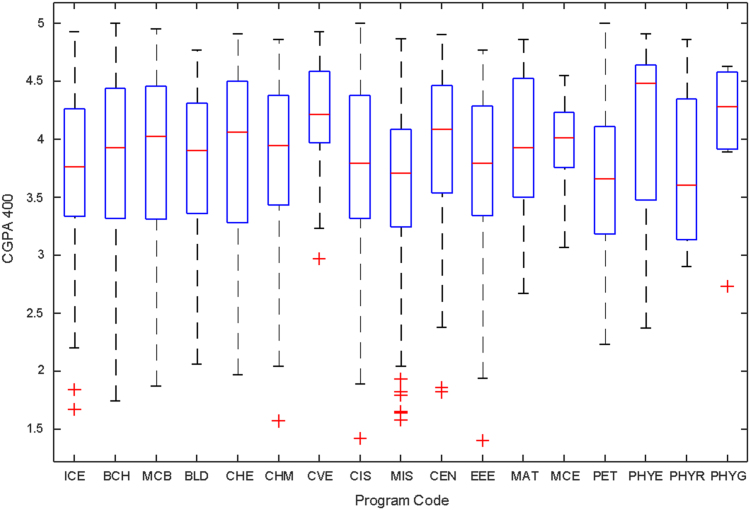
Boxplot of CGPA400 data for Female STEM students (2010–2014).

**Fig. 10:**
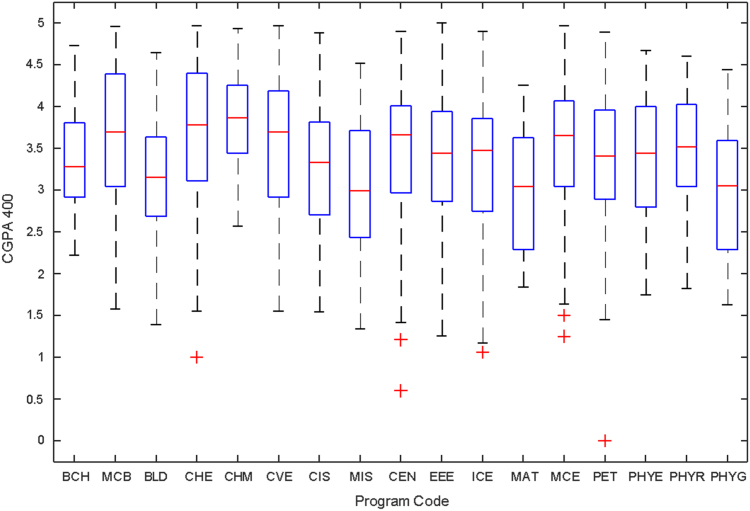
Boxplot of CGPA400 data for Male STEM students (2010–2014).

**Fig. 11:**
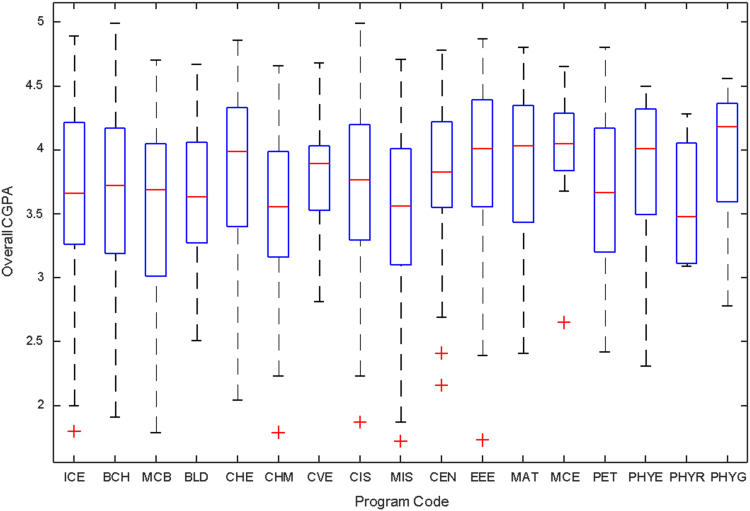
Boxplot of Overall CGPA data for Female STEM students (2010–2014).

**Fig. 12:**
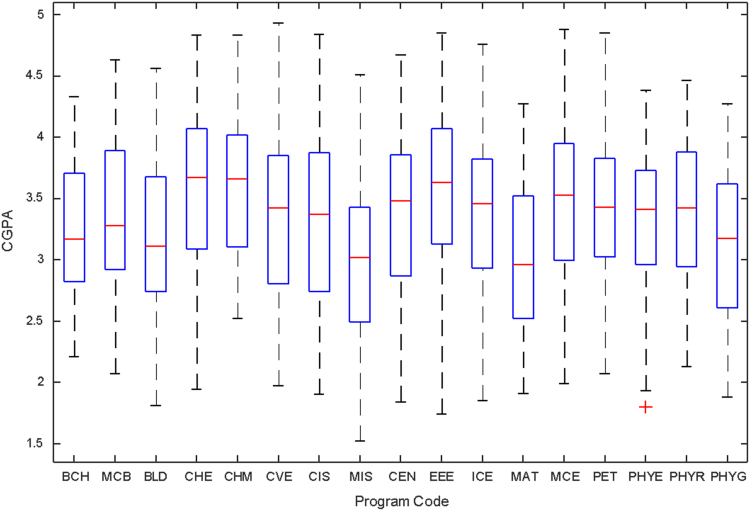
Boxplot of Overall CGPA data for Male STEM students (2010–2014).

Data of male and female undergraduate students was retrieved from the Students department of records and the Center for systems and information services at Covenant University. The data signposts the cumulative grade point average at the end of the secondary education (SGPA) and cumulative grade point averages from the first to the fourth year of study (CGPA 100–CGPA 400) and the overall cumulative grade point average (CGPA). The boxplots of SGPA, CGPA 100, CGPA 200, CGPA 300, CGPA 400 and CGPA of Female STEM students is given in Fig. 1, Fig. 3, Fig. 5, Fig. 7, Fig. 9, Fig. 11 while the boxplots SGPA, CGPA 100, CGPA 200, CGPA 300, CGPA 400 and CGPA of Male STEM students is given in Fig. 2, Fig. 4, Fig. 6, Fig. 8, Fig. 10, Fig. 12.
